# Artificial intelligence role in advancement of human brain connectome studies

**DOI:** 10.3389/fninf.2024.1399931

**Published:** 2024-09-20

**Authors:** Dorsa Shekouh, Helia Sadat Kaboli, Mohammadreza Ghaffarzadeh-Esfahani, Mohammadmahdi Khayamdar, Zeinab Hamedani, Saeed Oraee-Yazdani, Alireza Zali, Elnaz Amanzadeh

**Affiliations:** ^1^Student Research Committee, School of Medicine, Shiraz University of Medical Sciences, Shiraz, Iran; ^2^Student Research Committee, Alborz University of Medical Sciences, Karaj, Iran; ^3^Student Research Committee, Isfahan University of Medical Sciences, Isfahan, Iran; ^4^Student Research Committee, Iran University of Medical Sciences, Tehran, Iran; ^5^Student Research Committee, Islamic Azad University of Karaj, Karaj, Iran; ^6^Functional Neurosurgery Research Center, Shohada Tajrish Comprehensive Neurosurgical Center of Excellence, Shahid Beheshti University of Medical Sciences, Tehran, Iran

**Keywords:** connectome, artificial intelligence, machine learning, neurological diseases, deep learning, neuroimaging

## Abstract

Neurons are interactive cells that connect via ions to develop electromagnetic fields in the brain. This structure functions directly in the brain. Connectome is the data obtained from neuronal connections. Since neural circuits change in the brain in various diseases, studying connectome sheds light on the clinical changes in special diseases. The ability to explore this data and its relation to the disorders leads us to find new therapeutic methods. Artificial intelligence (AI) is a collection of powerful algorithms used for finding the relationship between input data and the outcome. AI is used for extraction of valuable features from connectome data and in turn uses them for development of prognostic and diagnostic models in neurological diseases. Studying the changes of brain circuits in neurodegenerative diseases and behavioral disorders makes it possible to provide early diagnosis and development of efficient treatment strategies. Considering the difficulties in studying brain diseases, the use of connectome data is one of the beneficial methods for improvement of knowledge of this organ. In the present study, we provide a systematic review on the studies published using connectome data and AI for studying various diseases and we focus on the strength and weaknesses of studies aiming to provide a viewpoint for the future studies. Throughout, AI is very useful for development of diagnostic and prognostic tools using neuroimaging data, while bias in data collection and decay in addition to using small datasets restricts applications of AI-based tools using connectome data which should be covered in the future studies.

## Introduction

The human connectome describes a model of neuronal connections in the human brain from single neuron scales to macroscale brain networks. It utilizes various neuroimaging modalities to identify the relationship between the structural and functional connectivity of the brain not only in people with healthy brains but also in patients with neuropsychological disorders (Betzel and Bassett, 2017; Contreras et al., 2015; Elam et al., 2021).

The main research fields about connectomes include understanding the complex network architecture of the human brain, the key characteristics of the structural and functional connectomes, the effects of structural changes on brain function, and the importance of connectomes on the diagnosis and prognosis of different neurological and psychological diseases. Finding the human brain connection tracts and the mechanisms of brain functioning have been considered important and challenging issues over the decades (Pospelov et al., 2019; Schmahmann et al., 2007). In this regard, the Human Connectome Project (HCP) was proposed in 2009 by a research team as an innovation in neuroscience research; and finally launched in 2010 with funding provided by the National Institutes of Health (NIH). This project aimed to map human brain connections in healthy adults to develop a dataset for investigating the relevance between human brain circuits and their functions (Elam et al., 2021; Van Essen et al., 2013). The turning point of HCP advent is related to developments in the field of neuroscience in the late 20th century; including the advancement in magnetic resonance imaging (MRI) technology such as structural MRI, resting-state functional MRI (fMRI), and diffusion MRI (dMRI) and the motivation of understanding nervous system pathways diagram [3]. In the following, launching HCP led to efforts to set up projects in the field of finding connectomes in disordered states (Tozzi et al., 2020).

Artificial intelligence (AI) has attracted the attention of the world as a widely used technology that simulates the human brain processes. Recently, the importance of using AI has been evaluated in various aspects of life sciences including medicine (Lee et al., 2017). Machine learning (ML) and deep learning (DL) are two approaches of AI that are applied to the medical field, especially medical imaging as clinical decision support systems. It seems that the field of medical imaging is one of the most suitable areas for investigating the application of AI (Castiglioni et al., 2021). Numerous studies have been conducted on the evaluation of the structural and functional connectome data associations based on ML and DL models in various neurological and psychiatric diseases in different aspects from early diagnosis to prediction of treatment outcomes. Based on our knowledge, no systematic review has been conducted on the usage of AI for the analysis of connectome data and the development of related models. However, it seems necessary to review recent progressions in this field to find gaps in this field and make it possible to fill them in future studies. Therefore, the objective of this systematic review is to peruse the articles related to ML and DL-based models and algorithms on human connectome data.

## Methods

### Search strategy

In this systematic review, we conducted a thorough search using PubMed, Google Scholar, and Embase, employing MESH and Emtree keywords including “connectome” AND “AI” OR “machine learning” OR “deep learning” in English. PRISMA guideline was used for screening and filtering the collected studies (Page et al., 2021). We aimed to identify peer-reviewed papers exploring the relationship between connectome and advanced computational techniques, specifically machine learning and deep learning. The screening process involved a sequential assessment of titles, abstracts, and full texts, with two independent authors ensuring the reliability of the selection and information extraction. Discrepancies were resolved collaboratively. This systematic approach enhances the credibility of our review, providing a concise synthesis of the current state of knowledge at the intersection of Connectome and advanced computational methodologies.

Inclusion criteria included (1) the use of connectome data, (2) subjects were human, (3) the use of ML or DL for analysis of connectome data, (4) studies published on original data, (5) peer-reviewed papers; while the exclusion criteria included (1) the use of imaging data but no connectome data, (2) animal models studies, (3) lack of results related to accuracy of applied algorithms, (4) systematic and other review studies, (5) case studies, and (6) editorials.

## Results

### Study selection

**Figure 1:**
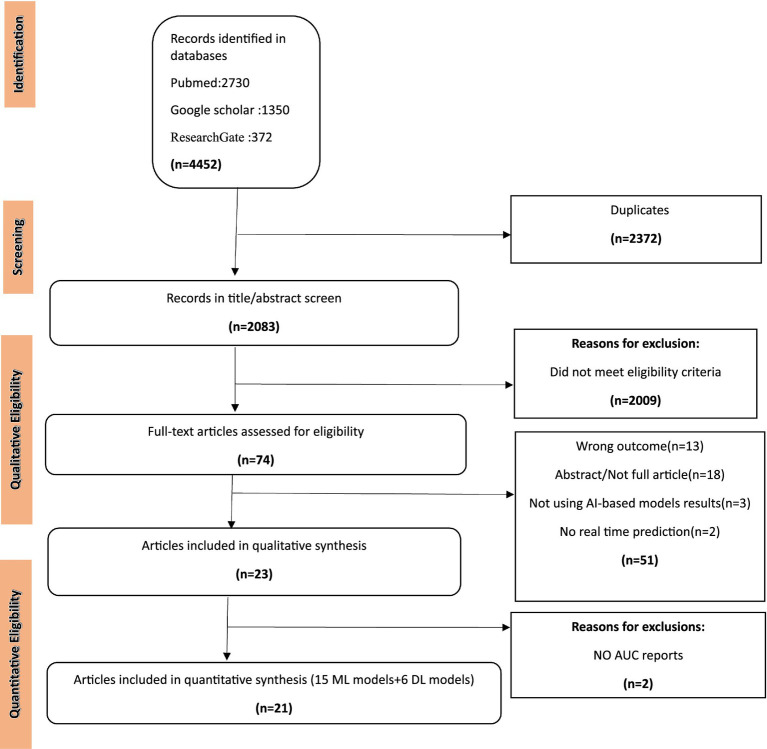
PRISMA flow diagram. ML, machine learning; DL, deep learning.

A total of 4,452 articles were collected from three databases and 2,372 articles were removed due to duplicates. After removing duplicates and checking references for additional articles, a total of 2,083 articles were screened, while 2,009 articles did not meet the eligibility criteria because they were not research studies, no AI-based methods were used, no connectome data were used, and no full-length article was available. In addition, 74 full-text articles were assessed with more details among which 51 articles were removed due to not reporting results of AI-based models. Next, 2 articles were reviewed among which because of not using AI-based models for data analysis (Figure 1).

The papers for this study were chosen if they had effectively explored the relationship between the connectome and various ML methods. Original research, retrospective, cross-sectional, and cohort studies were the types of studies considered eligible for this systematic review. The study did not target specific patient conditions; however, any research mentioning connective tissue disorders and utilizing ML methods was deemed acceptable. Every study, encompassing ML, DL, and neural network approaches, was included. This comprehensive approach aimed to capture the diverse applications of artificial intelligence in understanding the connectome and associated disorders. Finally, 22 articles were included in the present systematic review.

### Machine learning-based models and algorithms on connectome data

In recent years, several models have been developed to predict various phenomena in neural diseases using ML algorithms reviewed here. ML techniques could explore the relationship between brain connectome patterns, complex human traits, and polygenic architecture. A summary of studies that developed ML methods on connectome data has been provided in Table 1. In the study by Maglonac et al., fMRI-based static and dynamic temporal synchronization between large-scale brain network nodes was used to predict complex traits, such as fluid intelligence, educational attainment, and dimensional measures of anxiety, depression, and neuroticism. Besides, they predicted age, sex, and polygenic scores. They found that beyond age and sex, connectome-based features could effectively predict complex traits including fluid intelligence and educational level. Interestingly, the mentioned traits were negatively related to static brain connectivity in the frontal area (Maglanoc et al., 2020).

**Table 1:** Application of ML models on connectome data for specific aims.

S. No.	Author		Machine learning technique	Study design	Target condition
1	Gleichgerrcht et al. (2020)	2021	Single-layer feed-forward neural network classification model	Training cohort and three other sites as a testing cohort	Binary surgical outcome of patients with drug-resistant temporal lobe epilepsy
2	Ma et al. (2020)	2020	RVR*	Cross-sectional study	Short-term/acute insomnia and chronic insomnia
3	Payabvash et al. (2019a)	2019	Naive Bayes, random forest, SVM with linear kernel, SVM with polynomial kernel, and neural networks*	Original study	The use of neuroimaging techniques to investigate white matter microstructure in ASD* in children.
4	Munsell et al. (2015)	2015	Elastic net, SCCA, SVM classifiers, and deep learning*	Two-stage connectome-based prediction framework	Temporal lobe epilepsy (TLE)
5	Sarwar et al. (2021)	2021	Deep neural networks	Mapping whole-brain structural and functional connectivity matrices for 1,000 healthy adults using diffusion-weighted and resting-state functional MRI data	Functional connectivity (FC) in the human brain based on structural connectivity (SC)
6	Munsell et al. (2019)	2019	SVR, OLSR*	analysis of language scores and brain connectomes	REFRACTORY temporal lobe epilepsy (TLE)
7	Bruin et al. (2023)	2023	Linear SVM model	Multi-site, cross-sectional study	Obsessive-compulsive disorder (OCD) functional connectome evaluation
8	Tymofiyeva et al. (2019)	2019	J48 pruned tree classifier,	Longitudinal study	Major depressive disorder (MDD) in adolescent treatment
9	Chen et al. (2022)	2022	MKL-SVM*	Retrospective, cross-sectional, multicenter study.	Subjective cognitive decline (SCD)
10	Payabvash et al. (2019b)	2019	Naïve Bayes, random forest, SVM with linear kernel, and SVM with polynomial kernel	Cross-sectional study	Auditory over-responsivity (AOR)
11	Maglanoc et al. (2020)	2020	Shrinkage linear regression	Cross-sectional study	Genetic basis of functional connectivity in the human brain
12	Zhang et al. (2020)	2020	SVM	Cross-sectional study	post-traumatic stress disorder (PTSD)
13	Barile et al. (2022)	2022	SVM, Random Forest	Original research	Classification of multiple sclerosis (MS) based on grey matter connectome
14	Chen et al. (2020)	2020	Logistic Regression, Decision Tree Classifier, and XGBoost*	Original study	“Chemo-brain” or cognitive impairment in breast cancer patients who have undergone chemotherapy
15	Hannum et al. (2023)	2023	LDA, ANN, SVM, NC, CORR*	Original study	classify cognitive states based on functional connectome data obtained from fMRI scans

* ASD, autism spectrum disorder; MKL-SVM, multiple kernel learning support vector machine; RVR, multivariate relevance vector regression; SVR, support vector regression; OLSR, ordinary least squares linear regression; SCCA, sparse conical correlation analysis; LDA, linear discriminant analysis; ANN, multi-layer perceptron neural network; NC, nearest-centroid; CORR, correlation-based.

The researchers developed ML models to analyze the relationship between functional and structural patterns in the human brain. They aimed to explore how well the structural connectivity could predict its functional connectivity by using diffusion-weighted and resting-state functional MRI, respectively. Eventually, by applying deep learning networks, they perceived that structural connectome could explain significant differences between individuals in cognitive performances (Sarwar et al., 2021). This relation was also to predict serious problems in different diseases. Another study guided by Munsell et al. investigated the relationship between the architecture of neural networks and naming performance in patients suffering from temporal lobe epilepsy (TLE; Munsell et al., 2019). They utilized a structural connectome-based approach and an ML model to assess language naming performance using T1-weighted and diffusion MRI scans. Their ML model accurately predicted the naming performance in patients with medication refractory TLE, while this function was mainly associated with the temporal and frontal areas. They highlighted that the ML connectome-based models could be a promising approach to gain insights into the neural mechanisms underlying language impairments in neurological disorders.

In addition, Gleichgerrcht et al. (2020) conducted a study, aimed to investigate the potential of using structural connectome hubs to predict surgical outcomes in patients with TLE. They employed ML models to investigate the association between structural network integration and postsurgical outcomes. They used preoperative MRI data of 121 individuals with medication-resistant TLE to train the neural network models. Further, they used a dataset from 47 independent TLE patients from other centers to evaluate the predictive value of the model. They demonstrated that the lateral and medial temporal regions were associated with surgical outcomes. Indeed, individuals with abnormal integration structural networks had less chance of becoming seizure-free. The results of this experiment were consistent with previous studies regarding network abnormalities in TLE. Munsell et al. assessed the effectiveness of different ML algorithms in predicting the postsurgical outcomes of patients with TLE using structural connectome data (Munsell et al., 2015). For this purpose, they applied various ML algorithms on the diffusion MRI scans of epilepsy cases. They realized that support vector machines (SVMs) and random forests (RF) achieved high accuracy in predicting the outcomes. Further, they identified specific features of the structural connectome, such as connectivity patterns in a few specific brain regions, which played a crucial role in the accuracy of outcome prediction. They mentioned that using ML algorithms based on structural connectome has had similar results to the clinical findings presented by expert clinicians about predicting the outcome after surgery in patients with epilepsy.

Prediction of treatment outcomes was not limited to surgeries. Cognitive behavioral therapy (CBT) is a first-line treatment for adolescent major depressive disorder (MDD). In a study, a supervised ML algorithm was applied to structural connectome to predict symptom reduction in depressed patients treated with CBT. They obtained structural connectome data from diffusion MRI scans of depressed patients before receiving CBT. Next, they indicated that specific connectivity patterns in structural connectome were associated with the prediction of more significant depressive symptom reduction in patients receiving CBT. Regarding these results, they claimed this model could be used as a promising predictor of CBT effectiveness in all adolescent patients with MDD (Tymofiyeva et al., 2019). Connectome data could be used to predict the outcomes of treatments in not only neural diseases but also in cancers developed in other parts of the body. As an example, Chen et al. conducted a study to investigate the application of structural and functional connectome features in ML models for predicting “chemo-brain” in women receiving chemotherapy for breast cancer. They aimed to identify specific brain connectivity patterns with biomarker potential for predicting chemotherapy-induced cognitive impairments. Finally, they achieved an accurate connectome-based prediction model, which predicts the cognitive outcome with non-invasive tools in breast cancer cases who receive chemotherapy (Chen et al., 2020). The identification of biomarkers in childhood diseases is one of the most important factors for prevention and early intervention or postponing the progression of diseases. For instance, the white matter connectome edge density is assumed an imaging biomarker in children with autism spectrum disorders (ASD; Payabvash et al., 2019a). In this study, they applied ML models focusing on the white matter connections using diffusion MRI data to evaluate the connectivity patterns in ASD patients. They studied 14 ASD children and 33 typically developed children and finally found less density of connectome edges in posterior white matter tracts of ASD children. They indicated the feasible ML algorithms based on the structural connectome for diagnosing ASD in children. Progressive neurodegenerative diseases are among the diseases for which the identification of biomarkers via non-invasive methods is immediately required. Chen and colleagues stated that ML models based on multimodal connectomes have predictive potential of preclinical stages of Alzheimer’s disease (AD). For this purpose, structural and functional MRI, and positron emission tomography (PET) were used to construct a multimodal connectome model. They revealed that multiple kernel learning-support vector machines (MKL-SVM) could distinguish between preclinical stages of AD and healthy controls with high accuracy, which can be a promising approach for timely interventions and personalized treatment strategies (Chen et al., 2022).

The use of structural connectome data was also used for uncovering neural mechanisms of other diseases. Payabvash et al. conducted a study to investigate the correlation of white matter connectome with auditory over-responsivity (AOR; Payabvash et al., 2019b). Indeed, they employed ML classifiers on the edge density imaging data from diffusion tensor imaging (DTI) and high-resolution T1 scans. This technique measures the density of connections between different brain regions to examine the white matter connectivity patterns in individuals with AOR. They identified specific edges in connections of the white matter connectome that were consistently altered in individuals with AOR compared to the healthy controls. These findings provide new insights into the neural basis of AOR and demonstrate the potential of edge-density imaging and ML models for identifying biomarkers for this sensory processing disorder. Grey matter connectome analysis using MRI data and ML models could provide an accurate classifier for different clinical profiles of multiple sclerosis (MS) patients. Indeed, it was demonstrated that ML classifiers are valuable tools for understanding the heterogeneity of MS and aiding in the accurate classification of different clinical profiles, which may have implications for personalized treatment and management strategies (Barile et al., 2022). Magnetoencephalography (MEG) connectome data also could be used in ML models for classifying individuals with post-traumatic stress disorder (PTSD). Researchers used these data and built an accurate model to differentiate combat-related PTSD cases from trauma-exposed controls based on their MEG connectome patterns. They highlighted the neural mechanisms underlying the disorder and paved the way for potential diagnostic applications (Zhang et al., 2020). This study showed that MEG connectome data and ML algorithms can be used in the classification of other mental disorders.

Noteworthy, the functional connectome data has been used in fewer studies than structural connectome; however, it seems to include valuable information related to various diseases. ML models were used to evaluate the association between sleep quality and functional connectome in patients suffering from insomnia (Ma et al., 2020). They studied 29 individuals with short-term insomnia and 44 chronic insomnia patients. They conducted fMRI imaging for patients measured their sleep quality with the Pittsburg sleep quality index (PSQI) and applied the vector regression model. Whole-brain regional functional connectivity strength was used for PSQI prediction and there were similarities and differences between the two groups of chronic and short-term insomnia that helped identification of underlying mechanisms for each group. Functional connectome was also used for obsessive-compulsive disorder (OCD) classification. An ML approach was employed on the resting-state functional MRI data from multiple sites as part of the ENIGMA-OCD consortium. Indeed, they used a meta-analysis of resting-state functional MRI scans from 1,028 healthy controls and 1,024 OCD patients, to assess the difference between these two groups in whole-brain functional connectivity at both network and regional levels. Finally, they realized that sensorimotor networks play a crucial role in OCD, and shed light on its role in existing pathophysiology (Bruin et al., 2023). In this regard, another study evaluated functional connectome fingerprints and used these fingerprints to decode cognitive states, such as memory or attention. For this purpose, they used fMRI scan data to develop an ML model and showed that these fingerprints were informative enough to decode cognitive states with high accuracy levels. This study highlighted the potential of ML approaches in characterizing individual brain connectivity patterns and provided insights into cognitive states which in turn can potentially open doors to personalized interventions and cognitive state monitoring in various domains such as clinical diagnosis and cognitive neuroscience research (Hannum et al., 2023).

### Deep learning-based models and algorithms on connectome data

Various studies have utilized deep learning algorithms alongside machine learning models to predict outcomes and complications, as well as provide early diagnosis, for neurological and psychological diseases. A summary of these studies has been provided in Table 2. A study was conducted with the aim of residual distortion reduction in connectome data using high-resolution diffusion MRI (dMRI) preprocessed by HCP—pipelines and DL. For this purpose, “Distortion Correction Net (DrC-Net)” as an unsupervised DL framework was applied. U-NET was used to identify latent features of fiber orientation distribution (FOD) images while a transfer network for the propagation of deformation features was used in the proposed method. The presented method was trained randomly on 60 samples out of 100 cases from the HCP dataset and then tested on the rest. It was found that the presented method had similar distortion correction performance on both the training and test datasets based on the two evaluation methods including the mean squared difference (MSD) of the fractional anisotropy (FA) and the angular difference of main fiber directions. This study revealed dramatic improvement related to susceptibility distortion correction in both evaluation methods. In line with this finding, it is possible to significantly improve the mapping of connections between brain circuits by employing DrC-Net in minimizing the residual distortions in connectome imaging data (Qiao and Shi, 2020).

**Table 2:** Integration of connectome data and deep learning algorithms.

S. No.	Author		Technique	Study design	Target condition
1	Banerjee et al. (2020)	2021	CNN combined with a relational reasoning model	Original article	Language impairments in children with focal epilepsy (FE)
2	Funke et al. (2018)	2018	3D U-NET	Original research	Connectome reconstruction
3	Gleichgerrcht et al. (2018)	2018	DNN	Retrospective study	Post-surgical treatment of partients with epilepsy
4	Qiao and Shi (2020)	2020	DrC-Net	Original research	Susceptibility distortion correction
5	Yasaka et al. (2021)	2021	CNN	Prospective study	Parkinson’s disease (PD)
6	Sarwar et al. (2020)	2020	CNN	Original research	Connectome mapping

*CNN, convolutional neural network; DrC-Net, DistoRtion correction net; DNN, deep neural network.

Recently, studies have shown the importance of the role of DL models in predicting the different outcomes after surgery in patients with epilepsy. A deep relational reasoning network was applied to connectome data obtained from 51 children with Frontal lobe Epilepsy (FE) using preoperative conventional diffusion-weighted imaging. It was aimed to evaluate expressive and receptive language scores for language impairment prediction and postsurgical seizure outcomes based on Clinical Evaluations of Language Fundamentals (CELF) scores and International League Against Epilepsy (ILAE) classification, respectively. The method used in this experiment was “dilated CNN + RN,” also known as dilated convolutional neural network (CNN) combined with a relation network (RN), and the accuracy of model was assessed on whole-brain connectome data. The dilated CNN + RN approach represented an improvement in both the prediction of language impairment and seizure outcomes (seizure freedom and seizure recurrency) after surgery. In this model, gradient-based regression/classification activation maps were utilized on preoperative DWI data and it was successful in determining complex and non-local connectivity patterns in the connectome matrix for outcome prediction. In addition, 5-fold cross-validation, the cross-entropy loss function, and the synthetic minority over-sampling technique (SMOTE—to avoid overfitting due to the small sample size) were used for this evaluation. According to the results, the presented model had better performance than other investigated models (like SVR, Lasso, MLR, CNN + MLR) in language impairment prediction (Banerjee et al., 2020).

A study guided by Gleichgerrcht and colleagues on 50 patients with unilateral temporal lobe epilepsy (TLE). They aimed to investigate the role of the DL-based method on whole-brain structural connectomes derived from preoperative T1-weighted MRI and DWI in predicting postoperative seizure outcomes in these patients compared to conventional clinical prediction. Postsurgical seizure outcome (classified in two situations, becoming seizure-free or having persistent seizures) was evaluated by researchers at least 1 year after surgery. According to the result of this study, positive predictive value and negative predictive value were considered as seizure freedom and recurrent seizure outcome after surgery. In conclusion, DL model based on connectome data was more accurate for classification of seizure outcomes than the classification model based on clinical variables (PPV = 88 ± 7% and NPV = 79 ± 8% in the deep learning classification model in contrast to less than 50% accuracy in classification model for clinical variables; Gleichgerrcht et al., 2018).

Various research groups have used DL algorithms to develop diagnostic tools for neurodegenerative diseases via connectome data. Yasaka and colleagues designed a study to determine neural circuits in patients with Parkinson’s disease (PD) using a DL model on parameter-weighted and number of streamlines (NOS) -based structural connectome matrices derived from dMRI. For this study, researchers analyzed a total of 230 cases, consisting of 115 individuals with Parkinson’s disease and 115 healthy controls. The researchers employed the gradient-weighted class activation mapping (Grad-CAM) technique to examine the connectome matrices of patients and identify the specific brain regions that the convolutional neural network (CNN) was targeting. The results showed that the use of a DL approach on some parameter-weighted structural matrices had a higher performance in distinguishing PD patients from healthy controls compared to the conventional NOS-based matrix. DL models trained by the diffusion kurtosis imaging (DKI)-weighted connectome matrix had a significantly better diagnostic performance than other connectome matrices for PD. Eventually, the destruction of neural connections between the basal ganglia on one side and the cerebellum on the contralateral side was observed by researchers in PD patients. Meanwhile, DL models showed robustness for discrimination between PD patients and healthy people via parameter-weighted connectome matrices (Yasaka et al., 2021). Sarwar and colleagues developed a framework called block decomposition and stitching (BDS) by DL and dMRI data to map structural connectome and also to improve the accuracy of the conventional connectome mapping pipelines used for streamlining tractography. This experiment utilized dMRI and rsfMRI data to evaluate the precision of structural connectivity matrices in relation to functional connectivity matrices. The results showed that when the proposed model was assessed in combination with the typical connectome mapping pipelines, it yielded a 20–30% improvement in the accuracy of connectivity matrices reconstruction. It was also found that the BDS model can increase the structure–function connectome correlation strength between diffusion MRI and functional MRI data compared to conventional tractography. Likewise, this model can integrate conventional connectome mapping pipelines for accuracy improvement (Sarwar et al., 2020).

## Discussion and conclusion

Integration of artificial intelligence (AI) techniques into human brain connectomics studies has led to significant advancements in recent years. AI has emerged as a powerful tool for data analysis, modeling, visualization, and interpretation, revolutionizing the field of brain connectomics. The present systematic review highlights the AI-based algorithms using connectome data for different diseases. In this discussion, we explore the substantial contributions of AI in advancing our understanding of human brain connectivity and highlight its strengths and weaknesses. Consequently, we have come to understand that predictive models can be created to classify neurological diseases into subcategories, identify symptoms for early diagnosis, and establish connections between functional activities, prediction of post-surgical outcomes, and changes in connectome patterns. Despite this progress, there remains significant untapped potential for utilizing connectome data to diagnose a wider range of mentioned applications for other neurological diseases. AI algorithms have played a crucial role in improving the efficiency and accuracy of data acquisition and preprocessing steps in brain connectomics research. Prediction of surgical outcomes is important for surgeons, especially neurosurgeons. We mentioned the connectome data as a source of predicting surgical outcomes for drug-resistant TLE patients (Gleichgerrcht et al., 2018; Munsell et al., 2019). It seems that connectome can be a non-invasive and informative data with a potential for prediction of surgical outcomes. Early diagnosis is pivotal in central nervous system (CNS) diseases. Moreover, the non-invasiveness of connectome-based testing should be highlighted, as well as its greater access to data compared to traditional blood tests or biopsies. This method enables direct data collection from the brain, allowing for the identification of localized biomarkers. In this regard, a few studies have suggested the great potential of connectome data to find biomarkers of diseases in terms of the prognosis of ASD in childhood (Payabvash et al., 2019a). In addition to the non-invasiveness of using connectome, it is worth noting that data availability is more considerable than blood tests or biopsies, while it provides data directly from the brain which can be mentioned as localized biomarker identification.

Cognitive disorders are among the most common diseases that mainly do not have any definite treatments. In addition to the alterations in neural circuits and molecular interaction, signaling pathways, and neuron–neuron connections, considerable changes have been found in brain structure–function connectivity. Accordingly, using connectome data and AI not only helps detect changes in the cognitive performances of patients with neural diseases, but its exploration is useful for finding positive and negative networks in OCD and targeted interventions (Bruin et al., 2023). Neurodegenerative diseases are progressive diseases that have a high prevalence in the elderly, and no treatment is capable of limiting their progression. However, since these diseases are multi-factorial and sporadic, it seems necessary to find risk factors and predict the risk of their appearance in early times. However, the use of connectome data integrated with AI algorithms helps differentiate between the preclinical and clinical stages of AD and diagnose this disease effectively using hybrid PET/MRI imaging data (H. Chen et al., 2022). Another application of employing AI for the analysis of connectome data is subtyping diseases such as PTSD. These studies showed the potential of connectome data for using brain structural data in this disease for diagnosis, subtyping, and prediction of cognitive performance (Zhang et al., 2020). In this regard, developing new databases for collecting connectome data is one of the main tasks for developers and stockholders in healthcare systems. On the other hand, integrating connectomics data with AI is the main challenge for developing prognostic and diagnostic models for clinicians. These models should represent high accuracy and reliability to use as assisting tools by physicians and surgeons. According to the results, support vector machine (SVM), logistic regression (LR), gradient boosting, and random forest were among the most frequently used ML models applied to connectome data. The mentioned algorithms demonstrated high accuracy in predicting and classifying applications, though mainly no cross-validation analysis was applied on external data to find the exact accuracy of these algorithms. On the other hand, CNN and U-Net were the most frequent DL algorithms used for the analysis of connectome data. Despite connectome data being an imaging dataset with significant potential for analysis using deep learning algorithms, there are very few studies utilizing these algorithms. Therefore, limited number of studies on neurodegenerative disease such as AD, PD, and MS, low number of studies on brain tumors, low number of studies using deep learning algorithms for connectome data analysis, and low number of studies on the identification of early diagnostic symptoms are the main limitations regarding the use of A-based algorithms for analysis of connectome data.

In total, connectome provides a database for studying diseases and conditions related to the brain connectivity while its alterations are the source of information related to the diseases. Studying these alterations and using them for prediction of clinical phenomena such as diagnosis, prognosis and response to treatment are among the main missions of AI.

## Future perspectives

AI is a growing technology that can serve various fields such as medicine for infinitive applications. Connectome data has been recently identified in brain sciences as an informative and non-invasive source for the exploration of structural-connectivity relationships between various parts of the brain. Connectome data contains multi-aspect data so that in some studies only structural changes and their relation to the functions have been studied, while in other studies networks developed by connectivity of different parts or thickness-derived adjacency have been used as extracted data for more detailed studies (Chen et al., 2020; Payabvash et al., 2019b).

Hereby, we suggest using the more novel aspects of connectome data for studying other diseases, using studies with a higher number of imaging data to develop more robust AI applications using connectome data to replace invasive methods for diagnosis and prognosis of diseases with the use of connectome data.

In conclusion, the integration of AI techniques has revolutionized human brain connectomics studies, providing powerful tools for data analysis, modeling, and interpretation. AI has contributed to our understanding of brain connectivity, identification of biomarkers, and personalized diagnostics for neurological and psychiatric disorders. By leveraging AI’s capabilities, we can advance the field and pave the way for improved patient care.

## Data Availability

The original contributions presented in the study are included in the article/supplementary material, further inquiries can be directed to the corresponding author.
